# Atorvastatin associated with decreased hazard for death in COVID-19 patients admitted to an ICU: a retrospective cohort study

**DOI:** 10.1186/s13054-020-03154-4

**Published:** 2020-07-14

**Authors:** Guillermo Rodriguez-Nava, Daniela Patricia Trelles-Garcia, Maria Adriana Yanez-Bello, Chul Won Chung, Valeria Patricia Trelles-Garcia, Harvey J. Friedman

**Affiliations:** 1 Department of Internal Medicine, AMITA Health Saint Francis Hospital, Evanston IL, 355 Ridge Ave, Evanston, 60202 IL USA; 2 Department of Internal Medicine, John H. Stroger Jr. Hospital of Cook County, Chicago, IL USA; 3 Critical Care Units, AMITA Health Saint Francis Hospital, Evanston, IL USA; 4 University of Illinois College of Medicine, Chicago, IL USA

**Keywords:** COVID-19, Emerging disease, Adjuvant therapies, Critical care, Atorvastatin, Statins, Immunomodulators

To the editor,

Since the World Health Organization (WHO) declared the coronavirus disease 2019 (COVID-19) a pandemic, the medical community started a race against time to find effective treatments for this disease [1]. Atorvastatin as adjuvant immunomodulatory therapy is of particular interest given its low cost, known safety profile, and availability. The severe acute respiratory syndrome coronavirus (SARS-CoV) has been shown to interact with Toll-like receptors on the host cell membrane, increasing the expression of the MYD88 gene, ultimately activating NF-κB and triggering inflammatory pathways. Experimental models have demonstrated that statins stabilize MYD88 levels after a pro-inflammatory trigger, and, in a murine model, atorvastatin significantly attenuated NF-κB activation [2]. Furthermore, in the real world, two retrospective cohort studies reported a reduced risk of influenza death among statin users [3, 4]. Therefore, we assessed whether statin users at a dose of 40 mg daily had reduced inpatient mortality hazard from COVID-19.

In this retrospective cohort study, we used a de-identified dataset that included 87 adult patients with laboratory-confirmed COVID-19 admitted to our community hospital intensive care unit (ICU) located in Evanston, IL, from March to May 2020. We performed a Cox proportional hazards (PH) regression model to examine the relationship between adjuvant treatments and inpatient mortality. To minimize confounders, we adjusted for age, hypertension, cardiovascular disease, invasive mechanical ventilation, severity according to the National Institutes of Health criteria (respiratory rate > 30, SpO2 < 94%, PaO2/FiO2 < 300 mmHg or lung infiltrates > 50%), number of comorbidities (as a continuous variable), and other adjuvant therapies (including hydroxychloroquine, intravenous steroids, azithromycin, tocilizumab, colchicine, and antibiotics), forcing these variables into the model. We also performed a sensitivity analysis calculating the *E* value (with the lower confidence limit) as described by VanderWeele et al. [5, 6] for the obtained point estimate. The *E* value is defined as the minimum strength of association on the risk ratio scale that an unmeasured confounder would need to have with both the exposure and the outcome, conditional on the measured covariates, to explain away a specific exposure-outcome association fully.

The median age was 68 years (interquartile range [IQR], 58–75 years), 56 (64.4%) were males, and 50 (57.5%) were skilled nursing facility residents. Of these patients, 39 (44.8%) were ultimately discharged from the hospital, median length of stay 13 days (IQR, 7 to 21 days), and 48 (55.2%) had died, median length of stay 9.5 days (IQR, 3 to 14.75 days), a statistically significant difference (*p* = 0.032 by Mann-Whitney *U* test). A total of 24 (61.5%) survivors received atorvastatin 40 mg daily compared to 23 (47.9%) of non-survivors (*p* = 0.20 by chi-squared). In the multivariable Cox PH regression model, atorvastatin non-users had a 73% chance of faster progression to death compared with atorvastatin users (when probability = HR/HR + 1) (Table 1). The *E* value for the point estimate was 3.29 and the *E* value for the lower confidence interval was 1.69, meaning that a confounder not included in the multivariable Cox PH regression model associated with atorvastatin use and inpatient mortality in patients with COVID-19 by a hazard ratio of 1.69-fold each could explain away the lower confidence limit, but weaker confounding could not.

**Table 1:** Multivariable Cox regression of target interventions for COVID-19

**Intervention**	**Adjusted HR**^**a**^	**95% CI**	***p*value**^**b**^
Atorvastatin	0.38	0.18–0.77	0.008
Steroids	1.93	0.81–4.59	0.136
Hydroxychloroquine	0.81	0.31–2.10	0.675
Colchicine	0.41	0.17–0.98	0.045
Tocilizumab	1.17	0.42–3.25	0.765
Azithromycin	0.49	0.20–1.23	0.132

*Abbreviations*: *CI* confidence interval, *COVID-19* coronavirus disease 2019, *HR* hazard ratio

^a^Adjusted for age, number of comorbidities, hypertension, cardiovascular disease, severity, invasive mechanical ventilation, and antibiotics other than azithromycin

^b^*p* < 0.05 was considered statistically significant

In conclusion, we found a slower progression to death associated with atorvastatin in patients with COVID-19 admitted to our ICU. Given the observational nature of this study, results should be taken with caution; randomized controlled trials are needed to confirm this benefit (STATCO19, identifier NCT04380402). To date, supportive care remains the mainstay of therapy, and the clinical efficacy for various treatments is still under investigation.

## Data Availability

The datasets used and analyzed during the current study are available from the corresponding author on reasonable request.

## Abbreviations

CI: Confidence interval
COVID-19: Coronavirus disease 2019
HR: Hazard ratio
ICU: Intensive care unit
IQR: Interquartile range
PaO2/FiO2: Arterial oxygen partial pressure to fractional inspired oxygen
PH: Proportional hazards
SARS-CoV: Severe acute respiratory syndrome coronavirus
SpO2: Oxygen saturation
WHO: World Health Organization
